# Co-evolution between an Endosymbiont and Its Nematode Host: *Wolbachia* Asymmetric Posterior Localization and AP Polarity Establishment

**DOI:** 10.1371/journal.pntd.0003096

**Published:** 2014-08-28

**Authors:** Frederic Landmann, Jeremy M. Foster, Michelle L. Michalski, Barton E. Slatko, William Sullivan

**Affiliations:** 1 Department of Molecular, Cell and Developmental Biology, Sinsheimer Labs, University of California, Santa Cruz, California, United States of America; 2 Centre de Recherche de Biochimie Macromoléculaire, CNRS, Montpellier, France; 3 Molecular Parasitology, New England Biolabs, Ipswich, Massachusetts, United States of America; 4 Department of Biology and Microbiology, University of Wisconsin Oshkosh, Oshkosh, Wisconsin, United States of America; University of Liverpool, United Kingdom

## Abstract

While bacterial symbionts influence a variety of host cellular responses throughout development, there are no documented instances in which symbionts influence early embryogenesis. Here we demonstrate that *Wolbachia*, an obligate endosymbiont of the parasitic filarial nematodes, is required for proper anterior-posterior polarity establishment in the filarial nematode *B. malayi*. Characterization of pre- and post-fertilization events in *B. malayi* reveals that, unlike *C. elegans*, the centrosomes are maternally derived and produce a cortical-based microtubule organizing center prior to fertilization. We establish that *Wolbachia* rely on these cortical microtubules and dynein to concentrate at the posterior cortex. *Wolbachia* also rely on PAR-1 and PAR-3 polarity cues for normal concentration at the posterior cortex. Finally, we demonstrate that *Wolbachia* depletion results in distinct anterior-posterior polarity defects. These results provide a striking example of endosymbiont-host co-evolution operating on the core initial developmental event of axis determination.

## Author Summary

Filarial nematodes are responsible for a number of neglected tropical diseases. The vast majority of these human parasites harbor the bacterial endosymbiont *Wolbachia*. *Wolbachia* are essential for filarial nematode survival and reproduction, and thus are a promising anti-filarial drug target. Understanding the molecular and cellular basis of *Wolbachia*-nematode interactions will facilitate the development of a new class of drugs that specifically disrupt these interactions. Here we focus on *Wolbachia* segregation patterns and interactions with the host cytoskeleton during early embryogenesis. Our studies indicate that centrosomes are maternally inherited in filarial nematodes resulting in a posterior microtubule-organizing center of maternal origin, unique to filarial nematodes. This microtubule-organizing center facilitates the concentration of *Wolbachia* at the posterior pole. We find that the microtubule motor dynein is required for the proper posterior *Wolbachia* localization. In addition, we demonstrate that *Wolbachia* rely on polarity signals in the egg for their preferential localization at the posterior pole. Conversely, *Wolbachia* are required for normal embryonic axis determination and *Wolbachia* removal leads to distinct anterior-posterior embryonic polarity defects. To our knowledge, this is the first example of a bacterial endosymbiont required for normal host embryogenesis.

## Introduction

The phylum Nematoda comprises up to 1 million species and is one of the most diverse and successful, with members colonizing all possible ecological niches on earth [1], [2]. Nematodes have an extraordinary ability to adapt to the parasitic life style [3]–[6] and as a result exert profound impacts on agriculture and human health. The Spirurina clade contains only animal parasites, among them the Onchocercidae or filarial nematodes [5]. These thread-like worms are tissue-dwelling parasites, transmitted by arthropods, usually black flies or mosquitoes, to all classes of vertebrates except fish. It is estimated that 150 million people are infected with filarial nematodes, with 1 billion living at risk in tropical areas. Filarial nematodes lead to debilitating diseases such as onchocerciasis (caused by *Onchocerca volvulus*) and lymphatic filariasis (*Brugia malayi, Brugia timori, Wuchereria bancrofti*) [7]. A total of eight species of filarial nematodes are responsible for these neglected tropical diseases. With the exception of *Loa* and certain *Mansonella* sp., all other human filariae harbor an alpha-proteobacterium of the genus *Wolbachia*. This symbiosis is restricted to the family of Onchocercidae among nematodes [7], [8]. In addition, *Wolbachia* are also widespread among arthropods [9] and the bacteria of this genus have been classified into different supergroups, as defined by MultiLocus Sequence Typing [10], [11]. The supergroups C and D represent the majority of *Wolbachia* in filarial species and are restricted to the Onchocercidae [8].

*Wolbachia* are required for filarial nematode fertility and survival [12] and we previously showed that removal of either supergroup C or D bacteria by antibiotic therapies against *O. volvulus* or *B. malayi* leads to extensive apoptosis [13]. Yet little is known about the actual basis of the mutualistic interaction. Genomic analysis and experimental studies suggest that *Wolbachia* may contribute to metabolic pathways absent or partially missing in the nematode host, including synthesis of riboflavin, nucleotides and hemes [14]–[16]. However, the recent publication of the *Loa* genome, a *Wolbachia*-free human filarial parasite, revealed no metabolic compensation for the lack of mutualistic endosymbionts, suggesting caution in drawing conclusions on the basis of the symbiosis from genomic studies [17].

In the vast majority of filarial species, *Wolbachia* are present in the hypodermal chords of both male and female adult specimens, and in the female germline [8]. This is achieved through both asymmetric segregation during the mitotic divisions and cell-to-cell migration [18]. Immediately following fertilization, *Wolbachia* concentrate at the posterior region of the embryo. *Wolbachia* first localize in the posterior germline precursor lineage by rounds of asymmetric segregation until the 12-cell stage. They then reach a hypodermal lineage, and from this subset of posterior hypodermal cells, the bacteria colonize the whole dorsal and ventral hypodermal syncytia during late larval development, spreading toward the anterior of the worm [18]–[20].

Here we focus on the rapid migration and concentration of *Wolbachia* at the posterior pole immediately during the oocyte-to-embryo transition in *B. malayi* as this is a key unexplored initial event determining the distribution of *Wolbachia* in adult tissues. We used *C. elegans*, the sole well-studied nematode, as a reference for the oocyte-to-embryo transition in *B. malayi*. Although phylogenetically distant, the free-living and parasitic species both belong to the secernentean nematodes, and share a very similar embryonic development [21] [22] [23] [1]. To identify host factors involved in *Wolbachia* asymmetric enrichment after fertilization, we first characterized the cytoskeleton of the *B. malayi* embryo. As described below, we discovered a posterior microtubule-organizing center (MTOC) in the unfertilized mature oocyte. This is in striking contrast to *C. elegans*, in which the MTOC originates from the sperm-derived basal body/centrosome and induces cytoskeletal asymmetries essential for proper anterior-posterior polarity establishment [24]. Thus centrosome inheritance and its role in anterior-posterior polarity determination are dramatically different in *C. elegans* and filarial *B. malayi*. This maternally-derived *B. malayi* posterior MTOC facilitates *Wolbachia* concentration in the posterior of the newly fertilized egg. Using immunofluorescence and recently developed RNA silencing techniques [25], we show that host dynein is required for *Wolbachia* posterior enrichment in the egg. In addition, *Wolbachia* posterior localization requires *B. m.* PAR-1 and PAR-3, the *B. malayi* orthologs of *C. elegans* polarity-determining proteins Ce PAR-1 and Ce PAR-3. Finally, we demonstrate that *Wolbachia* removal results in Anterior-Posterior polarity defects, demonstrating for the first time that *Wolbachia* plays an essential role in these early embryonic developmental events.

## Methods

### *Brugia malayi* material

Live specimens were obtained from the NIH/NIAID Filariasis Research Reagent Resource Center (www.filariasiscenter.org). To obtain *B. malayi* adults devoid of *Wolbachia*, infected jirds were administered tetracycline at 2.5 mg/ml in drinking water (water changed daily) for a period of six weeks, followed by a one week clearance period. While this treatment is enough to deplete *Wolbachia* from filarial nematodes, the tetracyclin itself does not affect the host gene expression, including mitochondrial genes, as demonstrated by microarray after treatment of A. viteae, afilarial species devoid of *Wolbachia* [16]. Untreated infected jirds were maintained in a similar fashion as a control. After the clearance period, adult worms were recovered from the peritoneal cavities into preheated (37°C) culture medium RPMI-1640 supplemented with 100 U/ml penicillin, 100 µg/ml streptomycin, 2 mM L-glutamine, 0.25 µg/ml amphotericin B, and 25 mM HEPES (GIBCO).

### Ethics statement

The Animal Research and Care Program at UWO follows regulations and guidelines established by the USDA Animal Welfare Act, Public Health Service Policy, and the Association for the Assessment and Accreditation of Laboratory Animal Care (AAALAC). The protocol followed has been approved by UWO IACUC. Protocol 0-03-0026-000252-11-22-11, “Oral Tetracycline Treatment of Mongolian Gerbils (*Meriones unguiculatus*)”. Approval Date: 11/22/11. Expiration Date: 12/9/14 AAALAC #: 001268.

### Antibody production

All the *B. malayi* genes have been identified as C. elegans orthologs by reciprocal BLAST using the NCBI protein BLAST tool (http://blast.ncbi.nlm.nih.gov.gate1.inist.fr/Blast.cgi)

Two peptides were designed for each of B.m. γ-tubulin and B.m. *Zyg-9* and were used together to immunize rabbits:

-B.m. gamma tubulin (gene ID: 6105932 Bm1_55245):

(VRETVQTYRNATKPDFIEIN) and (GSHALEKISDRFPKKLVQTY)

*-B.m. zyg-9* (gene ID: 6096160 Bm1_06160):

(MHKSNPLKPPAP)

(RSDRSSSRIGRNTHRSNSVSRDSS)

For Dhc-1 a single peptide was used (gene ID: 6103168 Bm1_41435):

(LGGSPFGPAGTGKTESVKAL)

**Figure 1:**
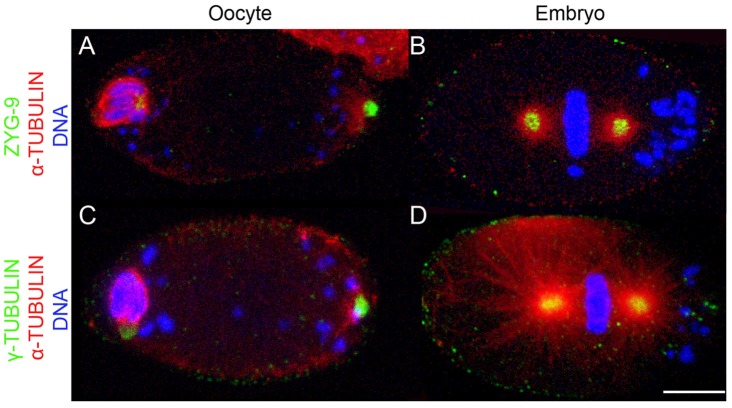
Unfertilized, mature *B. malayi* oocytes contain a polar MTOC. Mature oocytes (A,C) in meiosis I with bivalents associated with the anterior cortex, and embryos in first zygotic metaphase (B,D) are stained for DNA (blue), α-tubulin (red), and either the PCM marker Zyg-9 (A, B) or γ-tubulin (C,D) (green). In the oocyte, *Wolbachia* are associated with both poles and distributed in the cytoplasm. By the first zygotic division, *Wolbachia* are associated with the posterior pole. Scale bar = 5 µm.

Peptides were synthesized by the Organic Synthesis group of New England Biolabs with an additional N-terminal cysteine residue to facilitate conjugation to the carrier protein KLH using *m*-Maleimidobenzoyl-*N*-hydroxysuccinimide ester (MBS; Pierce, Rockford, IL) [21]. Sera were raised in rabbits by Covance Immunology Services, Denver, PA. Peptides were purified essentially according to a published procedure [26]. Antibodies raised against pericentriolar markers (i.e. B.m. gamma tubulin and B.m. zyg-9) co-localize with MTOCs (cf. Fig. 1). In both Spirurida (i.e. *B. malayi*) and Rhabditina (i.e. *C. elegans*), chromosomes are holocentric [27]. In *B. malayi*, the anti B.m. dhc-1 concentrates along the holocentric chromosomes during metaphase as dynein does in *C. elegans* [28].

### hsiRNA experiments

All the silencing experiments were performed as already described [25]. Briefly, *B. malayi* females were soaked in 1 µM of heterogenous short interfering (hsi)RNA mixtures for 48 hours before egg and embryo collection and fixation. PCR primers used to generate the primary dsRNAs contained T7 promoter sequence followed by two guanine bases at their 5′ ends for transcription by T7 RNA polymerase and enhanced transcription yield.

*-Par-1*(gene ID: 6100834 Bm1_29690)

*forward*: 5′- TAA TAC GAC TCA CTA TAG GGG AGA GGA ATC TTG CCA ACG G -3′

*reverse*: 5′- TAA TAC GAC TCA CTA TAG GGA ACT GCT TGT GCA GAT GCG C -3′

*-Par-3*(gene ID: 6103110 Bm1_41135)

*forward*: 5′- TAA TAC GAC TCA CTA TAG GGT TCT GGA TCC CGA TGA TCA G-3′

*reverse*: 5′- TAA TAC GAC TCA CTA TAG GGT AGA CGT GAT TTC CTA GCG G-3′

*-Dhc-1* (gene ID:6103168 Bm1_41435)

*forward*: 5′- TAA TAC GAC TCA CTA TAG GGA GCA ACT GTC AAG GAA AAG -3′

*reverse*: 5′- TAA TAC GAC TCA CTA TAG GGA TGG AGA CAA GTC GAT ATC C -3′

### Immunofluorescence and microscopy

Embryos were collected, fixed and stained as already described in detail [25]. Polyclonal anti B.m. Zyg-9, anti B.m. gamma tubulin and anti B.m. Dhc-1 were used at a dilution of 1∶100. Microtubule stainings were performed using the monoclonal DM1α antibody raised against α-tubulin (Cell Signaling Technology, Danvers, MA, USA) at a dilution of 1∶100. Cy5 goat anti-rabbit IgG and Alexa Fluor 488 goat anti–mouse IgG antibodies were used at 1∶150 (Invitrogen). Primary and secondary stainings were both performed overnight either at 4°C or room temperature. Actin stainings were performed using the fluorescent Atto 488 phalloidin (Sigma) at a dilution of 1∶100, added with secondary antibodies. The *Wolbachia* were visualized with propidium iodide (PI). We previously showed that the PI puncta only correspond to *Wolbachia* DNA by colocalization with *Wolbachia*-specific antibodies [19]. For propidium iodide (Molecular Probes) DNA staining, embryos were fixed then incubated overnight at room temperature in PBS+RNAse A (15 mg/mL, Sigma), in rotating tubes overnight. PI incubation itself was done after the secondary antibody wash (1.0 mg/mL solution) by simply shaking the eppendorf for 10 seconds in PBS followed by a 5 minute wash. 30 second centrifugations at 4000 rpm in between steps are enough to pellet all embryos. They were then mounted into Vectashield (Vector Laboratories, Burlingame, CA).

Confocal microscope images were captured on an inverted photoscope (DMIRB; Leica Microsystems, Wetzlar, Germany) equipped with a laser confocal imaging system (TCS SP2; Leica) using an HCX PL APO 1.4 NA 63 oil objective (Leica) at room temperature. 3-D movies were generated using the Volocity 3D Image analysis software (PerkinElmer).

## Results

### Maternally derived centrosomes lead to peculiar microtubule architecture in the filarial egg

*Wolbachia* have been shown to rely on host microtubules, kinesin and dynein in insects to properly segregate to the posterior germline pole plasm during oogenesis [29], [30]. To establish whether or not *Wolbachia* transmission also depends on similar cytoskeletal interactions in filarial nematodes, the microtubule network was characterized during the oocyte-to-embryo transition. To follow the microtubules and pericentriolar material (PCM), anti-*B.m.* γ-tubulin and anti-*B.m.* Zyg-9 antibodies were generated (Cf. experimental procedures; Fig. 1).

In the free living nematode *C. elegans*, as in most animal species, centrosomes are degraded during oogenesis, prior to diakinesis [31], [32]. In inseminated females, the cellularized oocyte follows a meiotic maturation phase, under the control of a sperm major protein (MSP) released from the sperm prior to fertilization [33]. During maturation, the germinal vesicle migrates away from the MSP source, with its associated acentriolar spindle, toward the unpolarized oocyte cortex. Centrosomes have a paternal origin and are inherited upon fertilization. The sperm-supplied centrosome participates to establishment of A-P polarity in the zygote, and the entry point defines the posterior pole of the egg [34].

**Figure 2:**
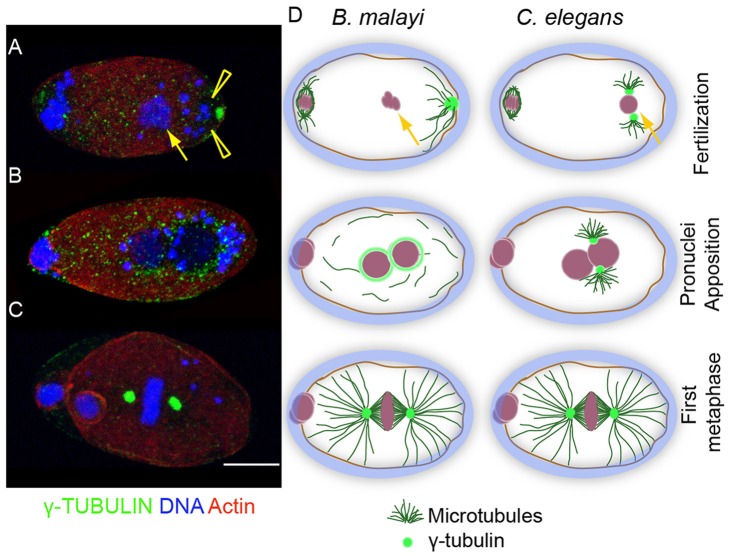
γ –tubulin dynamics during fertilization suggest a *de novo* origin of centrosomes in *B.malayi*. Fertilized eggs stained for actin (red), DNA (blue), and γ-tubulin (green). (A) Fertilization: the arrow points to the sperm in an egg in meiosis I, γ-tubulin foci (small green dots) are present throughout the cytoplasm. Arrowhead highlights *Wolbachia* (larger blue structures). (B) Pronuclei apposition. γ-tubulin foci concentrate around the pronuclei. Note the increased number of γ-tubulin foci compared to (A). (C) Metaphase: γ-tubulin foci form poles of metaphase spindle. Scale bar = 5 µm. (D) Comparison of zygote formation in *B. malayi* versus *C. elegans*. Yellow arrows point to the paternal pronuclei. In *B. malayi*, no MTOC is associated with the paternal pronucleus, while a cortical, maternal MTOC is present at the pole. γ-tubulin distributes around the pronuclei and precedes a *de novo* centrosome formation.

**Figure 3:**
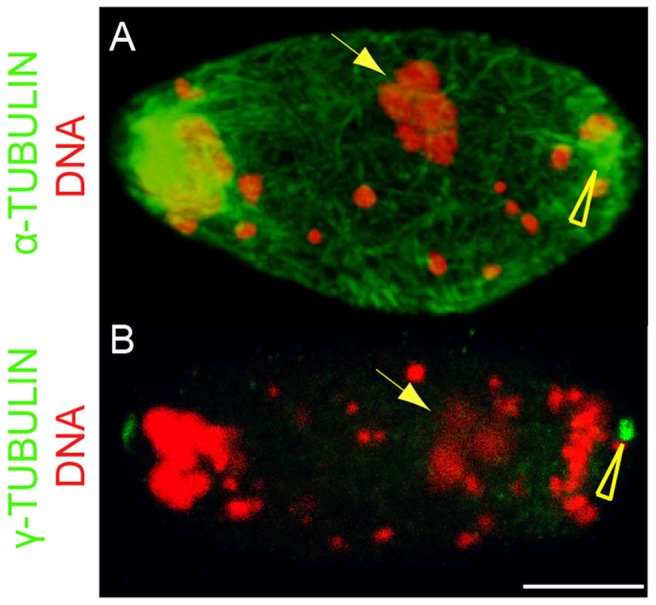
Absence of sperm-associated MTOC during fertilization. Two fertilized *B. malayi* eggs in meiosis I, stained for total DNA (red), and for either α –tubulin (A, green) or γ-tubulin (B, green). Arrows point to the sperm derived chromatin. Note in (A) the five paternal chromosomes still condensed. Arrowheads point to the maternal MTOC. There is no MTOC associated with the sperm-derived chromatin. All eggs are oriented with the anterior to the left, scale bar = 5 µm.

**Figure 4:**
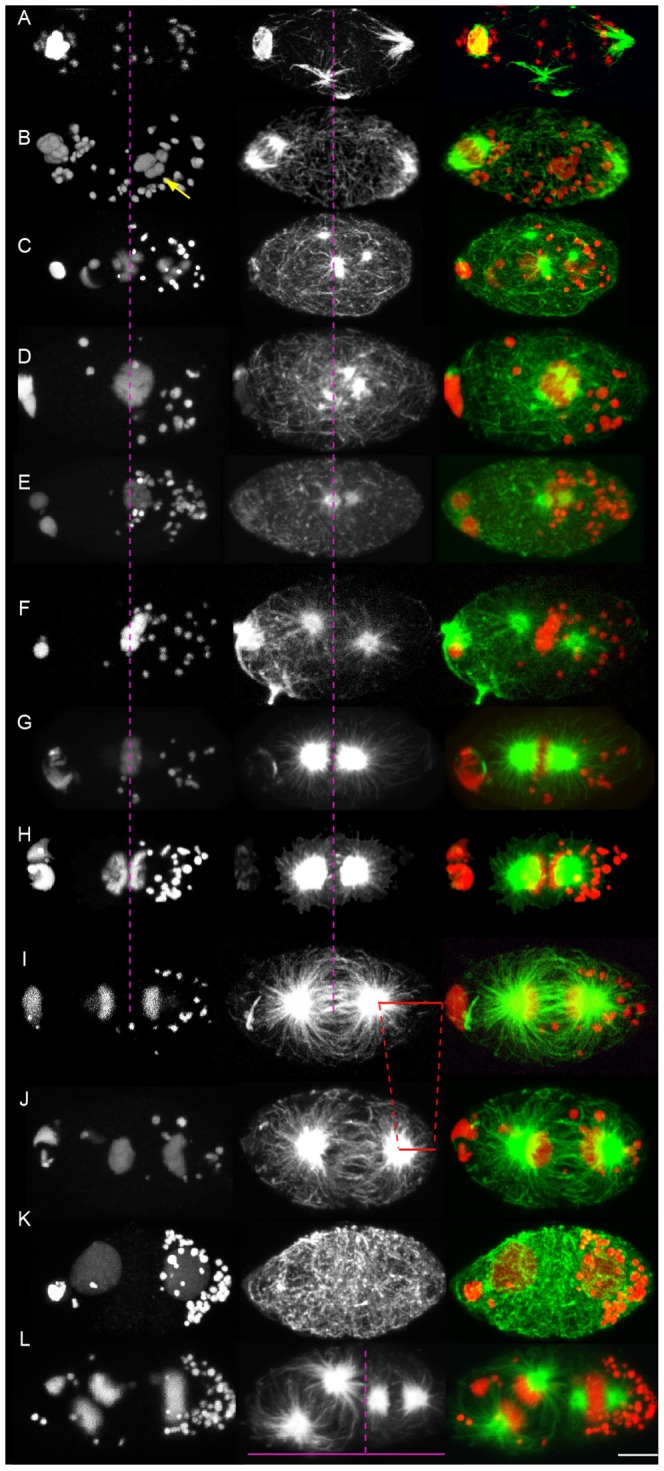
*Wolbachia* dynamics from fertilization to the two-cell stage in *B. malayi*. Whole mount eggs and embryos stained with propidium iodide to reveal the DNA (first column and red), and with an anti-α tubulin highlighting microtubules (second column and green). *Wolbachia* appear as DNA positive cytoplasmic foci (red). The dotted purple line highlights the equator from (A) to (I), and the asymmetry between blastomeres in (L). The red dotted line shows establishment of asymmetric spindle movement in late anaphase in (I) to (J). Anterior to the left, based on localization of polar bodies. (A) Prior to fertilization and (B) Fertilization (arrow points to the sperm/male pronucleus). (C) Pronuclei migration and condensation. (D) and (E) Prophase. (F) and (G) Metaphase. (H) Early anaphase. (I) and (J) Late anaphase. (K) Two-cell stage. (L) Two-cell stage in division. Scale bar = 5 µm.

In contrast to *C. elegans*, the presence of a microtubule-organizing center (MTOC), located at the opposite pole of the germinal vesicle was detected in unfertilized mature meiosis I oocytes from *B. malayi*. This polar MTOC is defined by both the presence of PCM components γ-tubulin and Zyg-9 proteins, and its ability to nucleate microtubules (Fig. 1, see also Fig. 2A, Movie S1). However, it disappears after fertilization, by the time pronuclei apposition occurs (Fig. 2(A) to (B)). Upon fertilization, no sperm-associated or paternal nucleus-associated MTOC was ever detected (Fig. 3(A) and (B), Movie S1; n>100). At this stage, microtubules do not nucleate at the surface of the paternal pronucleus, suggesting the absence of a paternally-derived MTOC. Rather, the anti-γ-tubulin antibody revealed numerous cytoplasmic foci (Fig. 2(A)). Some of these foci coalesce around the apposed pronuclei to form the MTOCs while the others are gradually degraded (Fig. 2(B) to (D)). This correlates with the microtubule dynamics at this stage (Fig. 4(C) to (E)). Together, these data demonstrate the presence in *B. malayi* of a MTOC-associated microtubule cytoskeleton in the mature cellularized oocyte, and suggest a maternal *de novo* origin of centrosomes in filarial nematodes, in contrast to *C. elegans* (Fig. 2 (E)).

### *Wolbachia* asymmetrically segregate in the zygote to concentrate in the posterior blastomere at the two-cell stage

**Figure 5:**
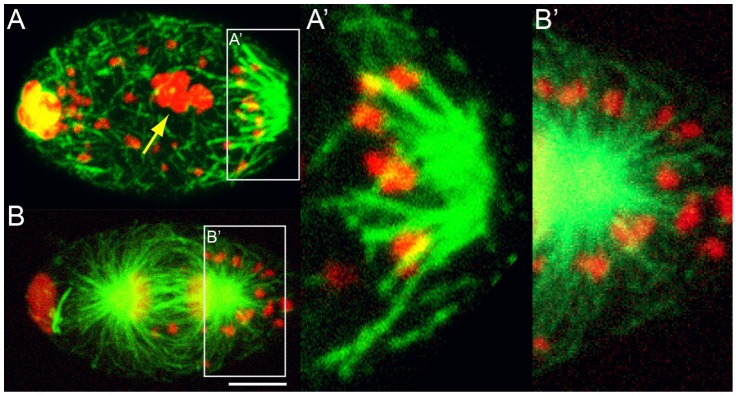
*Wolbachia* concentrate in the vicinity of host microtubules. *B. malayi* eggs soon after fertilization (A) or during the first anaphase (B), stained for DNA (red) and microtubules (green). In (A), the five paternal chromosomes are clustered in the center of the egg (yellow arrow), while meiosis I is being resumed. (A′) and (B′) are enlargements showing a close association between *Wolbachia* (red foci) and MTOC-derived microtubules (green). All eggs are oriented with the anterior pole to the left, scale bar = 5 µm.

We next examined *Wolbachia* dynamics in the mature oocyte and early embryo to better understand how they concentrate at the posterior blastomere during the two cell stage. We first characterized their dynamics in zygotes during the 1^st^ cell cycle (Fig. 4, n>100). Prior to, and soon after fertilization (Fig. 4(A) and (B)), *Wolbachia* are dispersed in the egg, sometimes showing a preference for the meiotic spindle and the opposite pole [19] (see also Fig. 5). The concentration in the posterior half of the egg starts during pronuclei migration and apposition (Fig. 4C), and is achieved by the beginning of prophase (Fig. 4D). This localization is maintained through mitosis (Fig. 4(D) to (J)) and enables the vast majority of endosymbionts to segregate in the posterior blastomere P1 after cytokinesis (Fig. 4K). This posterior segregation pattern is repeated in the dividing two-cell embryo (Fig. 4L). Thus, *Wolbachia* are asymmetrically localized very early in the zygote, to become enriched at the posterior end before entry into mitosis.

### *Wolbachia* are often observed near microtubules and as found in *Drosophila* oocytes, may physically interact with microtubules via motor proteins

We established that *Wolbachia* asymmetrically localize in the egg prior to the first mitosis, and are maintained at the posterior pole during mitosis. To further investigate a possible role of the microtubule cytoskeleton in *Wolbachia* dynamics, we looked for close association between the endosymbionts and microtubule network (Fig. 5, n>100). We found *Wolbachia* in the vicinity of microtubules emanating from the polar MTOC after fertilization (Fig. 5(A) and (A′)). Later during mitosis, we found *Wolbachia* organized along the posterior astral microtubules (Fig. 5(B) and (B′)). These data suggest that the microtubule cytoskeleton may be used by *Wolbachia* first for concentration, second for maintenance at the posterior pole of the egg.

### A dynein-based mechanism to concentrate *Wolbachia* to the posterior of the egg

**Figure 6:**
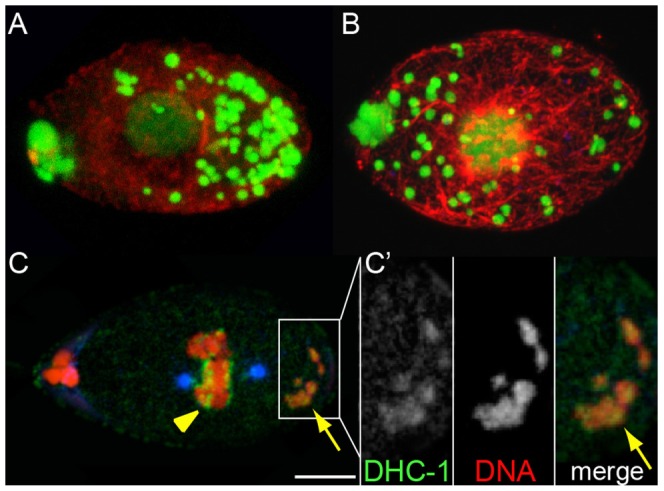
Host dynein is required for *Wolbachia* posterior concentration. Zygotes extracted after 48 hr-*in vitro* culture of (A) control adult females, or (B) *B.m.* dhc-1 hsiRNA treated adult females stained for DNA (green) and α-tubulin (red). In hsiRNA-Dynein knockdown embryos, *Wolbachia* fail to concentrate at the posterior pole, but rather occupy randomly the egg cytoplasm. (C) Zygote in metaphase stained for DNA (red), and with an anti- *B.m.* dhc-1 antibody (green). (C′) Enlargement of the posterior pole in (C) as indicated by the white box. Arrowhead points to the chromosome-associated dynein; arrow to the dynein co-localized with *Wolbachia*. Scale bar = 5 µm.

In *Drosophila*, *Wolbachia* rely on plus and minus end directed motor proteins for their concentration at the posterior pole of the *Drosophila* embryo [29], [30]. Our finding that *Wolbachia* closely localize to microtubules suggests they may concentrate at the posterior pole through their association with microtubule based motor proteins. The polar MTOC projects microtubule plus-ends inward and it was of interest to ascertain whether or not the *Wolbachia* may use the host minus-end molecular motor Dynein to segregate to the future posterior pole of the egg. To achieve this, the *B.m.* Dynein heavy chain 1 (*B.m.* Dhc-1) was silenced by soaking adult females in hsiRNA for 48 hrs [25], (Fig. 6). We collected a vast majority of multinucleated 1-cell eggs, as a result of chromosome segregation and cytokinesis failure when Dynein was reduced or absent. These highly penetrant phenotypes indicates that the hsiRNA is efficiently knocking down the Dynein levels. In these eggs, *Wolbachia* were evenly distributed in the cytoplasm (cf. Fig. S1). To circumvent the lack of developmental timing information in these eggs, we focused on zygotes prior to entry into the first mitosis (n = 10). In wild-type eggs, the majority of bacteria are at the posterior pole (n>100). In contrast, upon *B.m.* dhc-1 hsiRNA treatment, they no longer distribute asymmetrically (Fig. 6(A) and (B)).

To test a putative direct interaction between *Wolbachia* and Dynein, we raised an antibody against the *B.m.* dhc-1. Similar to studies in *C. elegans* [35], the anti-Dynein antibody decorates the condensed chromosomes in the zygote (Fig. 6C arrowhead). Significantly Dynein also colocalizes with posterior localized *Wolbachia* (Fig. 6(C) and (C′), arrow). This strongly suggests that *Wolbachia* may use the host Dynein and the polar MTOC for their initial asymmetric enrichment.

### A-P polarity determinants are required for *Wolbachia* maintenance in the posterior pole of *B. malayi* embryos

In *B. malayi*, after pronuclei apposition, the polar MTOC is no longer present in the egg. What then keeps *Wolbachia* in the posterior until the first division takes place? We tested the influence of Anterior-Posterior (A-P) polarity establishment in *Wolbachia* localization and maintenance.

Establishment of A-P polarity has been extensively studied in zygotes of the free living nematode *C. elegans*. In this species, symmetry breaking is triggered by sperm entry [34]. A remodeling of the cortical cytoskeleton is associated with a redistribution of the PARs polarity cues, as well as intense cytoplasmic streaming, to form an anterior and a posterior cortical domain by the beginning of mitosis. Subsequently, downstream polarity effectors are required to establish an asymmetric division [36].

**Figure 7:**
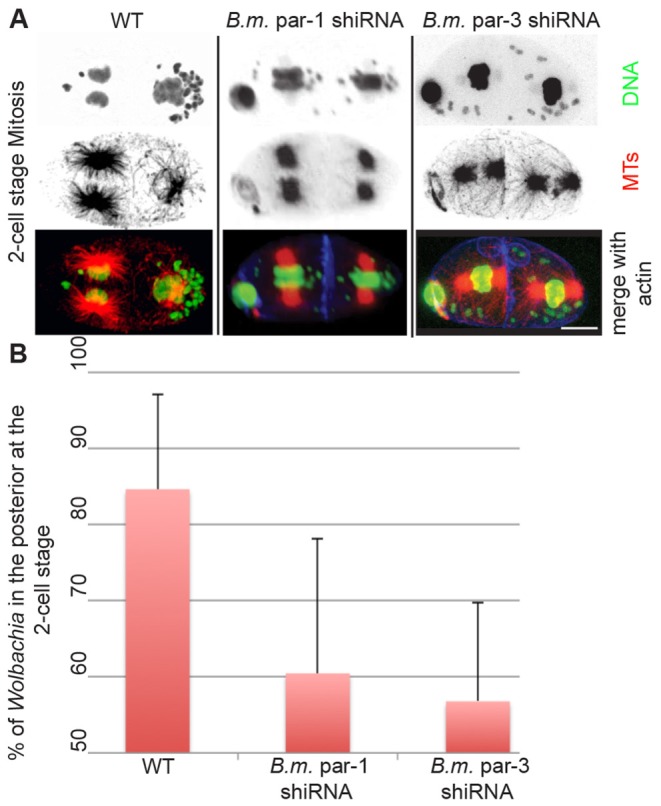
*B.m.* Par-1 and Par-3 are required for asymmetric segregation of *Wolbachia* in the two-cell embryo. (A) Two-cell embryos from either non-treated, or *B.m.* par-1 or par-3 hsiRNA-treated *B. malayi* females, stained for α-tubulin (“MT” red), DNA (green), and for actin (blue). Classic *C. elegans* par1 and par3 spindle rotation mutant phenotypes are produced. (B) Proportion of *Wolbachia* endosymbionts present in the posterior blastomere at the two-cell-stage (the posterior being defined whenever the polar bodies allow identification of AB and P1). For wild type embryos, n>100. For par-1 and par-3 hsiRNA-treated embryos showing division synchrony, n = 15. Scale bar = 5 µm.

To test whether PARs-induced symmetry breaking mechanisms dictate the bacteria asymmetric distribution, the *B. malayi* orthologs of *C. elegans* posterior PAR-1 and anterior PAR-3 were identified and silenced by hsiRNA. Due to the relatively low penetrance of the PAR-1 and PAR-3 hsiRNA phenotypes (∼30%, n>100 in both cases), we focused on dividing two cell embryos which showed classic PAR polarity-defect phenotypes: synchronous mitotic divisions and abnormal spindle orientation [37]. In wild-type *B. malayi* and *C. elegans* two-cell embryos, the anterior AB blastomere enters mitosis before the posterior P1 blastomere (Fig. 7A). This asynchrony is even more pronounced in *B. malayi*, where three-cell embryos, composed of AB daughters and dividing P1, are commonly observed. Also in *B. malayi*, like *C. elegans*, the posterior P1 spindle rotates by 90° to align along the A-P axis, while the AB spindle remains transverse (Movie S2). As in *C. elegans*, hsiRNA knockdown of either par1 or par3 disrupts the normal mitotic asynchrony between the two *B. malayi* blastomeres. In addition, upon *B. malayi* par-1 hsiRNA, the P1 spindle fails to rotate (Fig. 7A, Movie S3), while upon *B. malayi*. par-3 hsiRNA treatment, the AB spindle now rotates to align along the long (A-P) axis of the embryo (Fig. 7A). These timing and spindle orientation defects are strikingly similar to those observed in *C. elegans* [37] and reveal at least partial evolutionary conservation of functions for *B. malayi* PAR-1 and PAR-3. The presence of these polarity defects correlates with a loss of *Wolbachia* asymmetric segregation or maintenance at the posterior pole (Fig. 7B). This indicates that the A-P polarity determinants are essential for the stable enrichment of *Wolbachia* in the posterior P1 blastomere.

### *Wolbachia* are necessary for normal A-P polarity in *B. malayi*

**Figure 8:**
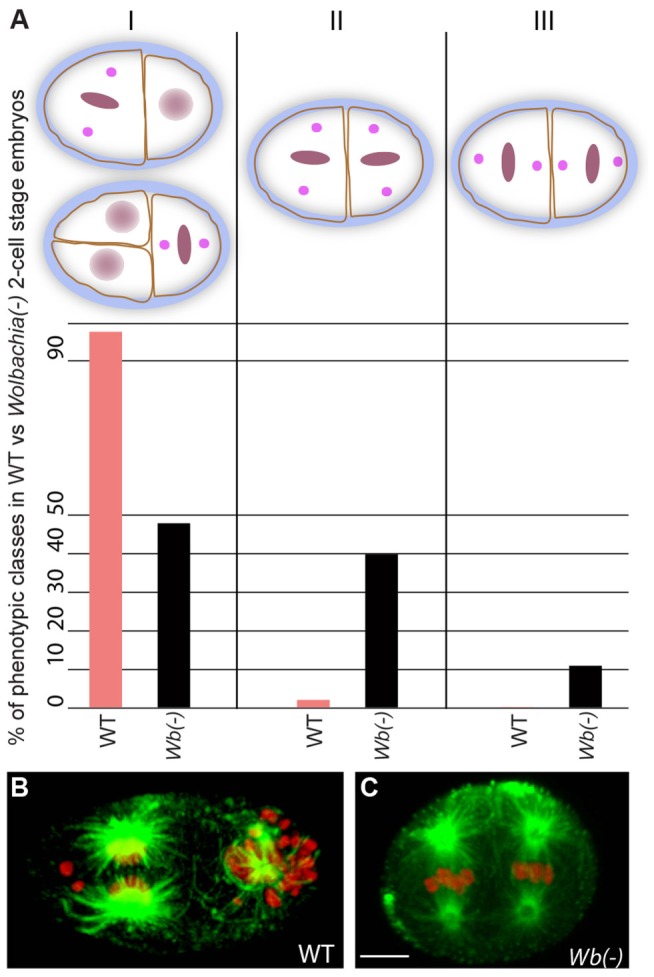
Loss of *Wolbachia* leads to A-P polarity defects. (A) Proportion of A-P polarity defects in dividing two-cell *B. malayi* embryos in presence (WT) or absence of *Wolbachia (Wb(-))*. Class I, normal, asynchronous division patterns. Class II, abnormal synchronous divisions and P1 spindle rotation failure. Class III, abnormal synchronous divisions and AB spindle rotation. (B) Wild-type and (C) *Wb(-)* embryos, stained for DNA (red) and α-tubulin (green). Scale bar = 5 µm.

By the first mitotic division, *Wolbachia* are predominantly concentrated in the posterior half of the *B. malayi* egg. In the *C. elegans* zygote, the complete establishment of the anterior and posterior cortical domains is already achieved by the beginning of mitosis [38]. As it is likely that A-P polarity set up in *B. malayi* takes place no later than in *C. elegans*, it was of interest to determine whether *Wolbachia* might influence the A-P polarity in the zygote. To investigate this, we analyzed A-P polarity in normal and *Wolbachia*-depleted two-cell embryos (cf. Experimental Procedures, [13]). This analysis yielded the following phenotypic classes (Fig. 8A): Class I included those with normal division patterns exhibiting mitotic division asynchrony and proper spindle orientation. Class II included those with “posterior polarity” defects exhibiting a failure of P1 spindle rotation and division synchrony, and Class III included those with “anterior polarity” defects exhibiting inappropriate rotation of the AB spindle and division synchrony. The vast majority of wild-type embryos (97%, n = 75) showed class I normal division patterns (Fig. 8(A) and (B)). Embryos devoid of *Wolbachia* (n = 27) displayed a dramatic loss of normal class I division patterns (48%). The remaining half of embryos lacking *Wolbachia* displayed either Class II posterior defects (40%, Fig. 8(B) and Movie S4) or Class III anterior (11%) defects. These results reveal that *Wolbachia* not only rely on A-P polarity cues for their posterior location but also are essential for proper establishment of AP polarity in its filarial nematode host.

## Discussion

### An unusual maternal origin of centrosomes and MTOCs in filarial nematodes

Centrosome inheritance is asymmetric in metazoan sexual reproduction. Usually, but not always, centrosomes are degraded in the female germline and provided paternally through the transformation of the sperm-derived basal body. This mechanism of inheritance ensures a tight control of centrosome number and MTOCs in the zygote, [39]. A dramatic exception to the typical pattern of paternal centrosome inheritance occurs in parthenogenetic development of unfertilized eggs in Hymenoperta. In this case, centrosomes and their associated MTOCS are derived exclusively from maternally derived components [40]–[42]. Our studies demonstrate a third unique centrosome/MTOC inheritance pattern in *B. malayi*. First, the unfertilized mature oocyte contains a maternal-derived MTOC. Second, despite fertilization, centrosomes appear to be produced *de novo* and to be maternally supplied. Accordingly, no paternally-derived MTOC was observed associated with the paternal chromatin after sperm entry.

Whether or not the maternal MTOC originates from a centrosome remains to be determined, since acentrosomal PCM has been shown to nucleate microtubules *in vitro* [43]. In any case, this maternal MTOC never interacts with the paternal chromatin and is degraded soon after fertilization. We find that during pronuclei apposition, the PCM component γ-tubulin accumulates around the nuclear envelopes as foci, and this correlates with microtubule enrichment at the nuclear surface. The presence of functional MTOCs capable of microtubule nucleation is only observed after entry of the pronuclei into mitosis. Together, these findings suggest centrosomes are derived exclusively from maternal components and perhaps form *de novo* in filarial nematodes. New centrosomal markers will be required to identify the origin and composition of the polar MTOC.

These findings also raise important questions regarding the mechanism of symmetry breaking and polarity establishment in filarial nematode embryos. In *C. elegans*, the paternally supplied centrosome and its associated MTOC play a crucial role in polarity establishment. The sperm derived centrosome/MTOC elicits a dramatic reorganization in the actomyosin cortical network and asymmetric localization of polarity components such as PAR-1 [34], [44]. It is currently unclear how much of a role the maternally-derived MTOC or fertilization plays in symmetry breaking and polarity establishment in the *B. malayi* embryo. The design of much needed new reagents suitable for filarial species will help us to understand the great variations on fundamental mechanisms between the free living *C. elegans* and filarial nematode species. This may help us to better understand peculiarities of the parasitic lifestyle, and sources of such evolutionary divergence.

### A role of host microtubules and dynein for *Wolbachia* posterior localization

In insects, *Wolbachia* must navigate the constantly changing cytoskeletal environment of the oocyte in order to concentrate at the posterior pole where the germline will form. *Wolbachia* rely on host microtubules for their transport through the oocyte. Early in oogenesis they rely on the plus-end motor protein kinesin. Later, the microtubules reorganize and reverse orientation requiring *Wolbachia* to engage dynein to complete their poleward journey. The studies presented here indicate that *Wolbachia* in *B. malayi* are also very likely to rely on microtubules and motor proteins for their asymmetric concentration in the posterior pole of the embryo. Unlike in *C. elegans*, prior to fertilization *B. malayi* oocytes possess a robust posteriorly positioned MTOC with microtubules emanating towards the anterior positioned meiotic spindle. Upon fertilization, the *Wolbachia* associate with microtubules and concentrate at this unusual posteriorly positioned MTOC. We also observe a striking co-localization between *Wolbachia* and the host dynein heavy chain. Significantly functional RNAi analysis demonstrates that dynein is required for this posterior enrichment. Thus in both insects and filarial nematodes dynein mediated movement is required for the asymmetric posterior positioning of *Wolbachia* to ensure germline incorporation.

Although the posterior MTOC is established prior to fertilization, fertilization is required for the posterior concentration of *Wolbachia*. We believe that the maternally supplied posterior MTOC contributes to the initial *Wolbachia* concentration at the posterior pole. However it appears that maintenance of *Wolbachia* at the posterior pole requires cytoplasmic rearrangements mediated by fertilization, such as the asymmetric cortical localization of PAR polarity cues, controlling an asymmetric dynein activity at the cortex.

### *Wolbachia* are transmitted to the posterior pole of the oocyte, the future site of germline formation

Unlike many intracellular bacteria, *Wolbachia* have no flagellum, and do not appear to rely on the actin cytoskeleton for intracellular transport. As with *Drosophila*, *Wolbachia* in *B. malayi* concentrate near, and perhaps associate, with microtubules [29]. Upon fertilization in *C. elegans*, the sperm brings a basal body giving rise to male pronucleus-associated MTOCs, establishing the posterior of the egg [34]. A similar mechanism in filarial nematodes would have explained an early, microtubule-based movement of *Wolbachia* toward the posterior of the embryo. However the fertilization mechanisms and remodeling of the cytoskeleton during this step appear dramatically different in *B. malayi*.

**Figure 9:**
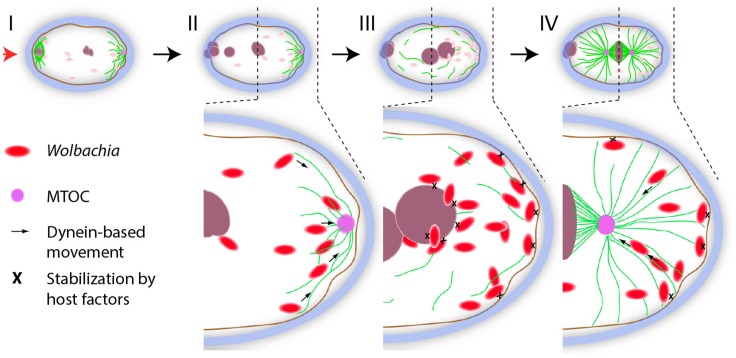
A model for *Wolbachia* asymmetric inheritance in the filarial egg. Schematic view of the key cytoplasmic and nuclear events and *Wolbachia* distribution after the fertilization (red arrowhead). I, fertilized egg in meiosis I; II, completion of meiosis; III, pronuclei apposition; and IV, mitosis.

Why is fertilization then needed to achieve the asymmetric enrichment, if the polar MTOC is already present in the oocyte? A simple model taking into account the microtubule cytoskeletal peculiarities of the filarial zygote could be envisioned (Fig. 9). A maternal polar MTOC projects microtubules inward, while meiosis is resumed at the opposite pole during fertilization (Fig. 9 I to II). In turn the polar MTOC is degraded and absent by the time pronuclei appose (Fig. 9 III), followed by entry into mitosis and set up of the mitotic spindle (Fig. 9 IV). After fertilization, when the meiotic spindle is no longer present, the bacteria concentration is preferentially displaced toward the MTOC. Cell cycle progression may also alter *Wolbachia* interaction with the Dynein complex, or its activation, resulting in more engagement on the microtubules [45]. At pronuclei apposition, *Wolbachia* are in the posterior compartment, most of them associated with the most posterior pronuclear envelope (paternal), but also in the cytoplasm, and in contact with the posterior cortex (Fig. 9 III, i.e. Fig. 2 (B) and (C)). The association with the nuclear membrane correlates with a perinuclear accumulation of γ-tubulin foci (Fig. 2C). Dynein is known to anchor the MTOC to the paternal nuclear envelope in *C. elegans* [46] [35]. This motor may play a role in centrosome biogenesis and recruitment to the nuclear envelope in *B. malayi*, and may also mediate this *Wolbachia* localization. Cortical dynein has also been shown to play a crucial role in spindle positioning in *C. elegans* [47], and *Wolbachia* cortical posterior localization could be mediated by the dynein itself. Once mitosis is triggered, whether *Wolbachia* interact with the astral microtubules or the cortex through dynein and/or other host factors, they remain trapped in the posterior compartment until cytokinesis occurs, and eventually segregate into the posterior blastomere (Fig. 9 IV, Fig. 4).

In the *C. elegans* two-cell stage embryo, symmetry breaking mechanisms similar to those observed in the zygote lead to a polarized P1 [24]. *Wolbachia* asymmetric pattern of segregation is perfectly repeated when P1 divides (Fig. 4L), confirming the importance of host A-P polarity signals in *Wolbachia* distribution in the early embryo. No polar MTOC is however required in P1 to achieve the same segregation observed in the zygote P0. It is interesting that *Wolbachia* has co-evolved to adapt to a microtubule dynamics and architecture unique to fertilization in filarial nematodes. This peculiar *de novo* centrosome inheritance raises many important questions regarding the filarial oocyte-to-embryo transition.

### *Wolbachia* are essential for establishing proper anterior-posterior polarity establishment in filarial nematodes

There are now a number of examples in diverse phyla in which bacteria have a profound influence on metazoan development [48]. For example, mice raised in a germ-free environment, exhibit defects in the enteric nervous system regulating gastrointestinal function [49]. Another striking example of animal bacterial interactions occurs in the Squid- *V. fischeri* symbiosis. The *V. fischeri* bacteria are required for proper development and morphology of the light organ of the squid. The bacteria induce very specific changes in cell size, morphology and microvilli formation [50].

Our analysis of the *Wolbachia-B. mayali* symbiosis provides a unique example in which the bacteria are required for normal host axis formation and embryonic development. *B. malayi* and *C. elegans* share similar division patterns during early embryogenesis, with AB dividing first, while in the posterior germline precursor P1, the spindle rotates to align along the long A-P axis. These traits are common among the nematode species so far examined [51]. Without *Wolbachia*, A-P polarity establishment is compromised in the filarial zygote, as revealed by division timing and spindle orientation defects at the two-cell stage, a hallmark of A-P polarity defects in nematode species.

How do the endosymbionts influence A-P polarity? Since *Wolbachia* concentrate to the posterior before mitosis in *B. malayi*, (a stage prior to establishment of A-P cortical domains in *C. elegans*), it is possible that *Wolbachia* directly influence localization and/or activation of *B. malayi* posterior polarity cues (i.e. PARs), or on downstream posterior polarity effectors. Conversely, our experiments silencing *B.m* par-1 and par-3, result in a failure of *Wolbachia* to become posteriorly enriched indicating that the PAR proteins are required for proper *Wolbachia* localization. In *Drosophila*, *Wolbachia* also associate with polarity determinants. *Wolbachia* closely associates with the Gurken polarity complex in the *Drosophila* oocyte and its titer regulated by Gurken levels. Significantly an overabundance of *Wolbachia* disrupts Gurken function [52].

The pioneering work of Sander in the 1950's demonstrated that displacing the ball of endosymbionts present in the leaf hopper *Euscelis plebejus* embryo from the posterior to a more anterior position produced ectopic posterior structures. This demonstrated a close association with posterior patterning determinants [53]. In nematodes *Wolbachia* not only rely on key host polarity factors for their germline transmission, but have become essential for the proper functioning of these determinants.

At this point, however, we cannot rule out a non cell-autonomous explanation for the effect of *Wolbachia*-depletion on host A-P polarity. Unlike in *C. elegans, B. malayi* embryogenesis takes place entirely in the female uterus, where the growth of the embryo is dependent on maternal nutrients acquired from the hypodermis [2], [19], [54]. In addition, the endosymbionts fill the hypodermal tissues, a major site for nutrient storage and metabolism in filarial nematodes, and this bacterial population is also cleared upon antibiotic treatment [13]. Thus, it is then possible that *Wolbachia* removal from the hypodermis leads to metabolic defects affecting a plethora of signaling pathways, including the embryonic polarity set up. A better understanding of symmetry breaking mechanisms in these parasitic nematodes will help us establish precisely how *Wolbachia* influence embryonic polarity.

In conclusion, we have shed light on the symbiosis mechanisms underlying *Wolbachia* transmission in the filarial embryo. They suggest a reciprocal dependence between the host and the symbiont starting as early as in the egg, explaining the success of antifilarial antibiotic therapies targeting *Wolbachia*, leading to massive embryogenesis defects.

## Supporting Information

Figure S1 **dhc-1 hsiRNA induces cytokinesis defects and results in multinucleated 1-cell eggs.** (A) Zygotes extracted after 48 hr-*in vitro* culture of *B.m.* dhc-1 hsiRNA treated adult females stained for DNA (green) and α-tubulin (red). In these multinucleated eggs due to cytokinesis failure, *Wolbachia* occupy randomly the egg cytoplasm.
(EPS)

Movie S1 **3D rotation of a fertilized** ***B. malayi*** **egg, showing nucleation of microtubules (green) at the opposite pole of the meiotic germinal vesicle.** The DNA stained with PI (red) shows the MTOC-free paternal chromatin in the center of the egg, during meiotic maturation. Note the close vicinity of some *Wolbachia* with the microtubules emanating from the polar MTOC.
(MOV)

Movie S2 **3D rotation of a fixed 3-cell** ***B. malayi*** **wild-type embryo, showing the microtubules (red), actin (blue) and DNA (green).** AB daughters are in the anterior. The posterior P1 contains the *Wolbachia*. Its spindle is aligned along the A-P axis.
(MOV)

Movie S3 **3D rotation of a fixed** ***B. m.*** **par-1 hsiRNA treated 2-cell** ***B. malayi*** **in division.** Microtubules (red), actin (blue) and DNA (green). The blastomeres are synchronous, and the posterior spindle do not align with the A-P aix but remains transverse. The *Wolbachia* no longer segregate asymmetrically.
(MOV)

Movie S4 **3D rotation of a fixed** ***B. malayi*** **2-cell stage embryo devoid of** ***Wolbachia*** **.** Microtubules (green) and DNA (red). The embryo displays a class II phenotype, with loss of division asynchrony and two parallel spindles.
(MOV)
